# Natural Causes of Sports- and Recreation-Related Deaths in the General Population: A 14-Year Review in Québec, Canada

**DOI:** 10.1016/j.cjco.2024.09.002

**Published:** 2024-09-10

**Authors:** Philippe Richard, Jérémie Sylvain-Morneau, Paul-André Perron, Philippe Joubert, Paul Poirier

**Affiliations:** a Direction de la sécurité dans le loisir et le sport, Ministère de l’Éducation, Québec, Québec, Canada; b Institut national de santé publique du Québec, Québec, Québec, Canada; c Bureau du coroner du Québec, Québec, Québec, Canada; d Institut universitaire de cardiologie et de pneumologie de Québec, Université Laval, Québec, Québec, Canada; e Faculty of Medicine, Université Laval, Québec, Québec, Canada; f Faculty of Pharmacy, Université Laval, Québec, Québec, Canada

## Abstract

**Background:**

This study analyzed trends in the frequencies and rates of natural deaths associated with sport and recreation activities in Québec, Canada, from January 2006 to December 2019, and investigated their etiology and characteristics.

**Methods:**

This descriptive retrospective study utilized data from coroner reports, as well as autopsy and police reports. Activity-specific incidence rates were calculated using participation data from the *Étude des blessures subies au cours de la pratique d’activités récréatives et sportives au Québec* (ÉBARS) and Canadian census population data.

**Results:**

A total of 297 natural deaths occurred, resulting in a population-based death rate of 0.26 per 100,000 person-years. The participation-based rate was 0.23 per 100,000 participant-years, focusing only on the 24 matching activities in both editions of the ÉBARS. Cycling (20.5%; n = 61), ice hockey (8.8%; n = 26), and hunting (8.1%; n = 24) were associated with the highest frequencies and rates of death. Most of the deaths (95.3%; n = 283) were of cardiac origin, with acute coronary syndrome and malignant cardiac arrhythmia identified as the most common causes. Automated external defibrillators were unavailable at 65% of death sites, and bystander cardiopulmonary resuscitation was performed in 60.9% of cases. Men accounted for the vast majority (92.6%; n = 275) of deaths. Death rates increased starting from age 35, peaking in males 45 and over.

**Conclusions:**

Efforts aimed at screening for cardiovascular risk factors and ensuring the widespread availability of automated external defibrillators at recreational sites, including remote areas such as hunting territories, could reduce the incidence of natural deaths associated with sport and recreation activities in Québec.

## Résumé

**Contexte:**

Cette étude visait à analyser les tendances quant à la fréquence et au taux de décès de cause naturelle associés à la pratique d’activités sportives ou récréatives au Québec, Canada, entre janvier 2006 et décembre 2019, et à examiner leurs causes et leurs caractéristiques.

**Méthodologie:**

Cette étude descriptive rétrospective porte sur des données issues de rapports de coroners, de même que de rapports d’autopsie ou de police. Les taux d’incidence selon les activités ont été calculés en fonction des données de participation provenant de l’*Étude des blessures subies au cours de la pratique d’activités récréatives et sportives au Québec* (ÉBARS) et du Recensement de la population canadienne.

**Résultats:**

Au total, 297 décès de cause naturelle sont survenus, soit un taux de mortalité de 0,26 par 100 000 années-personnes dans la population. Le taux fondé sur la participation était de 0,23 par 100 000 années-participants, en tenant compte uniquement des 24 activités communes aux deux éditions de l’ÉBARS. Le cyclisme (20,5 %; n = 61), le hockey sur glace (8,8 %; n = 26) et la chasse (8,1 %; n = 24) étaient associés à la fréquence des décès et au taux de mortalité les plus élevés. Dans la plupart des cas (95,3 %; n = 283), le décès était d’origine cardiaque; le syndrome coronarien aigu et l’arythmie maligne représentant les causes les plus fréquentes. Dans 65 % des endroits où un décès est survenu, aucun défibrillateur externe automatisé n’était accessible; une réanimation cardiorespiratoire a été réalisée dans 60,9 % des cas par un témoin. Les hommes représentaient la grande majorité (92,6 %; n = 275) des cas de décès. Les taux de mortalité augmentent à partir de 35 ans, atteignant un sommet chez les hommes de 45 ans et plus.

**Conclusions:**

Les efforts visant à dépister les facteurs de risque cardiovasculaire et à garantir la disponibilité généralisée des défibrillateurs externes automatisés là où les activités récréatives se déroulent, y compris dans les régions éloignées comme les territoires de chasse, pourraient permettre de diminuer l’incidence des décès de cause naturelle associés aux activités sportives et récréatives au Québec.

Engaging in regular exercise is beneficial for improving and preserving health.^1^^,^^2^ Consistent physical activity has been linked to a reduced risk of cardiovascular events, and physical fitness has been shown to contribute to a decrease in both cardiovascular-related deaths and overall mortality.^3^ These benefits can, however, be offset by fatalities of either natural or traumatic causes.^4^

In the province of Québec, Canada, 2234 deaths associated with sport and recreation activities were recorded from 2006 to 2019.^5^ Extensive research has been conducted to document the trends in the frequency and rates of the 1937 unintentional injury deaths that occurred during this period in the province.^5^ Still, 13.3% of the deaths (n = 297) associated with sport and recreation activities were due to natural causes and warrant further examination.^5^ In the realm of sports, a significant proportion of natural deaths stem from cardiac origins.^4^^,^^6^, ^7^, ^8^ Among athletes, sudden cardiac death (SCD) is the leading cause of nontraumatic fatalities.^9^^,^^10^ These deaths, although they are very infrequent, may arise from detectable hereditary or congenital heart conditions for which preventive screening strategies exist.^8^^,^^9^^,^^11^^,^^12^ Accordingly, approaches for screening athletes have been investigated in depth, with assessments of the pros and cons regarding the issue of screening, which often is reserved for high-level athletes.^9^^,^^11^^,^^12^ An important finding, however, is that most sports-related sudden deaths occur in nonathlete adults (including both regular men and women who engage in recreational sports, and men and women who are sedentary or recreationally active),^7^ populations which therefore also need to be included in studies designed to inform preventive initiatives.

Regarding this issue, in addition to the demographic, contextual, and activity-specific information that can be found in coroners’ investigation reports, autopsy reports document certain pathologies and anatomic peculiarities of victims of natural deaths.^7^ Moreover, police reports sometimes contain complementary valuable information, such as the proximity and incidence of use of an automated external defibrillator (AED).^7^ The combined examination of these complementary data sources may shed new light on the processes involved in developing and evaluating efficient prevention strategies relating to natural deaths in sports- and recreation-related settings. Therefore, this study examined the trends in the frequencies and rates of natural deaths associated with sport and recreation activities, for the January 2006-December 2019 period in the province of Québec, Canada. The study focused mainly on 3 parameters: (i) mortality numbers and rates based on sport and recreation participation data; (ii) mortality numbers and rates based on census data; and (iii) the etiology and characteristics of the fatalities.

## Methods

### Design and data source

Building upon a previous investigation,^5^ this retrospective descriptive study utilized data from the computerized database of the Bureau du coroner du Québec (CD-BCQ). This database holds standardized data for each mortality case, using a coding system based on the International Classification of Diseases, 10th revision (ICD-10). All 2234 cases related to sport and recreation activities (unintentional injury deaths and natural deaths) during the period from January 2006 to December 2019 in Québec, Canada had been identified previously^5^ through algorithms incorporating preselected keywords and ICD-10 codes, as well as specific procedures targeting cases coded subjectively by the BCQ as occurring "while engaging in sports" or "during participation in games or leisure activities."^5^ A meticulous screening process detailed in the data-flow diagram of a previous study also was carried out, which thoroughly outlined all information on inclusion and exclusion criteria.^5^ A double data-entry method was applied to separately document all fatalities associated with both sports and recreation activities. Investigators examined a spectrum of factors, including the specific activity associated with each fatality, the place of occurrence, the context, and whether alcohol or other psychoactive substances (nonalcohol drug information was sourced from coroners' reports) were consumed, as previously outlined.^5^ Activities included both sports played competitively and those played recreationally, as well as recreational activities that may not be defined consistently as sports in the literature (such as hiking, snowshoeing, and hunting). Recorded activities could be either organized or unstructured. For deaths to be included in the study, within the category “hiking and taking a walk,” the coroner's report needed to include specific descriptions, such as “hiking,” “trekking,” “taking a walk,” “walking for exercise purposes,” “leisure walking,” and “walking for fitness.” General demographic information, such as age at the time of death, gender, year of death, and the region of death, was extracted directly from the CD-BCQ.

Within the dataset of sports- and recreation-related deaths (n = 2234 cases), 1937 unintentional injury deaths were excluded from the present study, based on their ICD-10 codes (coded as V01 to X59), resulting in 297 deaths categorized under the generic code used by the BCQ to categorize natural deaths. The cause of death was confirmed further in the conclusion section of each report.^5^^,^^13^ In Québec, coroner reports are public records, according to the Coroners Act (chapter C-68.01),^14^ and thus, their use for research purposes did not require ethical approval.^5^ Authorization to use the accompanying documents (autopsy and police reports) for scientific research and publication purposes was granted by the chief coroner of Québec (June 27, 2022) and the Ministère de la Sécurité publique (November 21, 2022), following the provisions of article 31 of the law,^14^ under reference number 06595 (122787). All autopsies and expertise reports used in this study were conducted in accordance with the Coroners Act.^14^

### Data processing

The data-entry process, based on the coroner reports accompanying documents, occurred at the BCQ offices and was conducted by a board-certified cardiologist (P.P.). A board-certified pathologist (P.J.) also was involved, to clarify data elements in the autopsy reports. Information from all autopsy and police reports was entered directly into the database, which already contained the demographic, activity-specific, and contextual information associated with the 297 natural deaths related to sport and recreation activities. This entry process utilized the BCQ's file number to ensure that case-matching was accurate. Details about the availability of an AED and cardiopulmonary resuscitation (CPR) interventions were collected in the police reports and coronial reports, based on findings from investigations, interviews, and the evaluative judgment of the coroner or police officer. Autopsy reports provided patient data, including weight, height, and body mass index (BMI), along with heart-related macroscopic and histologic characteristics, comprising heart weight, left-ventricle thickness, interventricular septum thickness, presence of fibrosis, valve abnormalities, coronary malpositioning, coronary calcification, aortic anomalies, and fatty infiltration.^7^^,^^11^ The presence of the following cardiovascular risk factors (CVRFs) also was recorded: obesity (BMI >30 kg/m^2^, or obesity documented by the pathologist in the absence of BMI data); hypertension; dyslipidemia; smoking; diabetes mellitus; and a family history of cardiac diseases (sudden cardiac arrest, ischemic heart disease, or arrhythmic syndromes).^7^^,^^8^^,^^15^, ^16^, ^17^, ^18^, ^19^, ^20^ When available, information related to substances with potential cardiotoxic effects was sourced from coroners’ reports (derived from toxicological analyses). However, the data did not permit the classification of each substance into specific concentration thresholds, such as therapeutic, significant, or fatal levels. Therefore, these substances were defined as "potentially cardiotoxic substances" in the context of this study. All autopsy-related information in the final database was validated by an experienced pathologist (P.J.), with input from a cardiologist (P.P.). Causes of death, when available, were recorded directly from the autopsy report, and if unavailable there, from the coroner report. Additionally, causes of death recorded from the autopsy reports were cross-validated by another investigator (P.R.) using the coroner’s reports. Finally, a meeting was held with the 2 main investigators (P.R. and P.P.) to review and confirm the causes of all 297 deaths, utilizing cross-referenced information from autopsy and coroner's reports.

### Key definitions

Cardiac deaths were fatalities attributed to a cardiac cause, based on available evidence in the autopsy and coroner reports, as detailed elsewhere.^11^ Noncardiac deaths were those that did not meet the criteria for having cardiac-related causes.^7^ The reference definition used for sudden deaths, whether cardiac-related or not, was as follows: an unexpected event occurring abruptly, resulting in death within 1 hour from the onset of symptoms in witnessed cases, and within 24 hours in unwitnessed cases, based on available evidence.^2^^,^^7^^,^^9^^,^^11^^,^^21^ However, to account for logistic challenges in documenting the exact timing of deaths in unstructured settings, the study used a 24-hour window from the cessation of activity. Symptom onset in patients (who often were described as being unconscious and unresponsive) occurred while they were actively engaged in the activity, while they were pausing briefly, or when they had just finished. Given that emergency services and witnesses did not have the authority to declare death on the spot, and given that many events occurred in remote areas or in situations lacking a nearby medical authority, a broader 24-hour timeframe was necessary. This approach captured the full context of activity-related risks in real life and reflected the practical difficulties that can be encountered in documenting deaths in unstructured settings.

### Statistical analysis

The denominators used to calculate participation-based incidence rates were derived from the 2009-2010^22^ and 2015-2016^23^ editions of the *Étude des blessures subies au cours de la pratique d'activités récréatives et sportives au Québec* (ÉBARS [*Study of injuries sustained during sport and recreation in Québec*]).^5^^,^^13^ Values for missing years were estimated by assuming constant participation numbers, using 1 of the 2 ÉBARS editions: data from the earlier edition were applied for 2006-2014, whereas participation figures from the latter informed the data for 2015-2019, as previously described.^5^^,^^13^ The denominators for participation-based rates included only the 24 activities that were common to both editions of the ÉBARS, and numerators were calculated accordingly.^5^^,^^13^ In the current study, participation-based rates were calculated solely for activities, age groups, and sexes (also referred to as biological genders). The death count was divided by the participation figures of the ÉBARS, resulting in a rate per 100,000 participant-years. Population-based rates were calculated using age and sex estimates for Québec’s administrative regions, as of July 1 each year, obtained from the Institut de la statistique du Québec's website.^24^ These rates, presented per 100,000 person-years, reflect the annual death count relative to the population of the corresponding year. Therefore, the numerators in the population-based rates represent the total absolute numbers of unique sports- and recreation-related natural deaths. For both denominators, a high level of concordance was found between crude annual incidence rates, and age- (≤ 17, 18-44, 45-64, ≥ 65 years) and sex-adjusted annual incidence rates. As a result, only the crude rates are presented.^5^^,^^13^^,^^25^ Confidence intervals were computed at a 95% significance level, using the Gamma distribution method.^26^ Estimates with coefficients of variation > 33.3% were categorized as unreliable (indicated as "F" in Tables 1 and 2), in accordance with the Statistics Canada guidelines. Estimates with coefficients of variation ranging from 16.6% to 33.3% also were identified and necessitate careful interpretation.^22^^,^^23^

The years were categorized into 3 periods (2006-2009, 2010-2014, and 2015-2019), to enhance the precision of the estimated rates.^5^^,^^13^ Poisson regression was employed to investigate changes in rates over the period analyzed, for all activities, by estimating incidence rate ratios (IRRs).^5^^,^^13^^,^^27^ Periods were compared, to assess inter-period changes. IRRs were not computed for activities with < 5 deaths within a specific period. Analysis of linear trends, which reflects the average annual change in incidence rates between consecutive years, was also conducted. *P*-values were used to test the null hypothesis of no linear trend against the alternative hypothesis of a linear increase or decrease in the incidence rate at a significance level of 5%.^5^^,^^13^^,^^25^

Due to the limited number of cases for some causes of death, and to provide a specific perspective on cardiac deaths, significant differences between clusters of cardiac causes (acute coronary syndrome, malignant arrhythmias, and aortic causes) across age groups were assessed, using χ^2^ tests. Fisher’s exact test was applied specifically to aortic causes, owing to their limited number making a small sample size.^13^ To control for the overall type I error rate, a global test was conducted. If the global test yielded a statistically significant result, comparisons then were made among the 3 clusters of cardiac causes within their groups (eg, acute coronary syndrome vs the 2 other cardiac causes combined).

The analysis was conducted using SAS software (2019-2020, SAS Institute, Cary, NC). To assess the variance in participation-based rates, the working assumption was that denominator values remained stable, as the primary source of variability typically originated from the numerator, due to its having a lower value in most cases.^5^^,^^13^^,^^25^^,^^28^

## Results

### Participation-based rates

**Figure 1:**
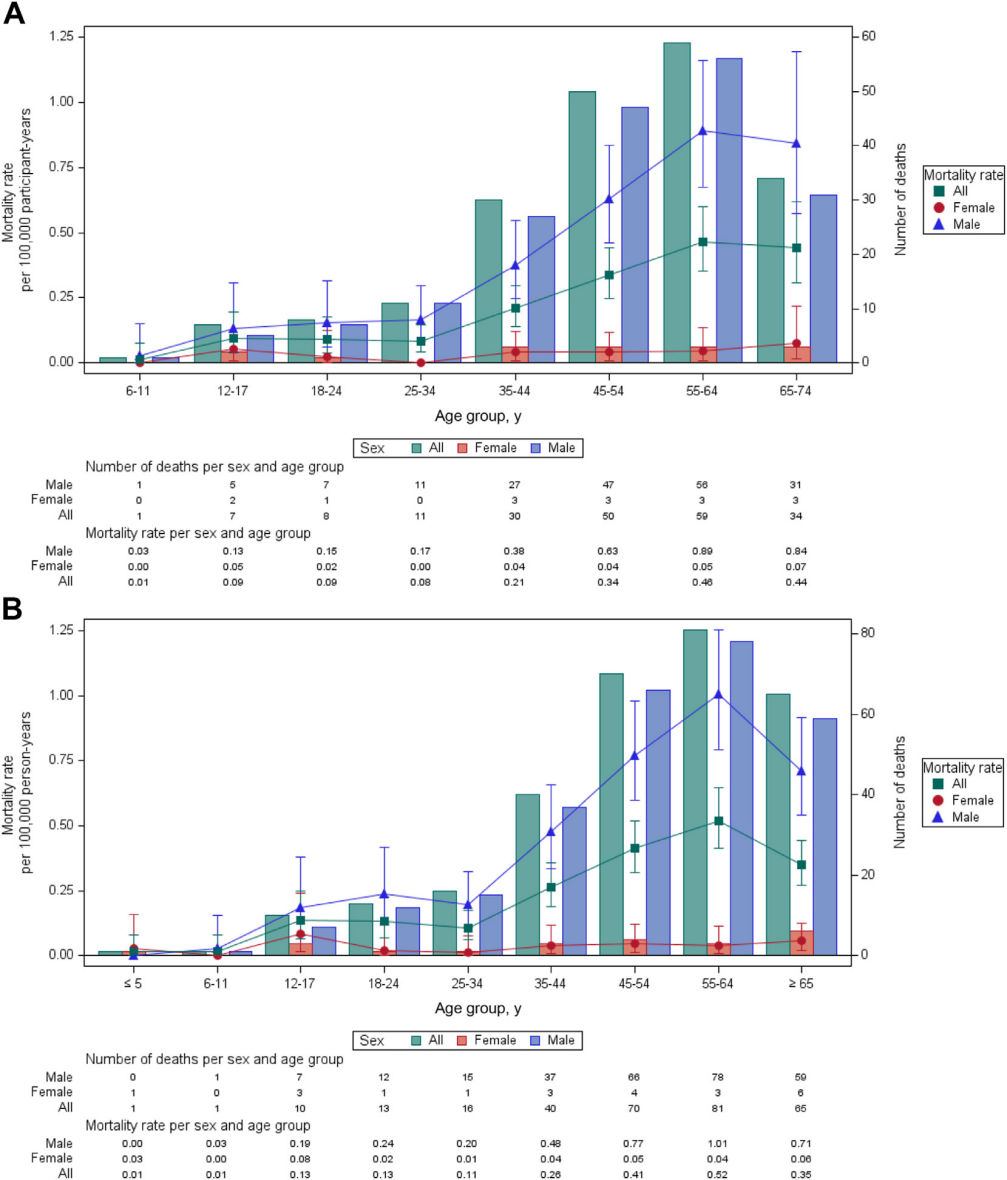
Numbers and rates for sport and recreation deaths by natural causes in Québec, Canada, by age group and sex, January 1, 2006, to December 31, 2019 (inclusive). (**A**) Numbers and participation-based rates considering populations surveyed in the *Étude des blessures subies au cours de la pratique d’activités récréatives et sportives au Québec* (ÉBARS). Rates are expressed as a number per 100,000 participant-years. (**B**) Numbers and population-based rates for all sports- and recreation-related deaths. Rates are expressed as a number per 100,000 person-years. Note that some of the rates presented do not comply with the diffusion criteria established by Statistics Canada regarding coefficients of variation (Supplemental Tables S3 and S4) and are provided for references purposes only.

From January 2006 to December 2019, the participation-based natural death rate in sport and recreation was 0.23 per 100,000 participant-years, considering only the 24 activities that were consistent in the 2 editions of the ÉBARS. Ice hockey had the highest participation-based rate, at 0.19 per 100,000 participant-years, followed by cycling, at 0.11 per 100,000 participant-years. There were no significant differences in IRRs for all activities (Table 1). Death rates increased starting from age 35 across all age groups, with the highest rates observed in males aged 45-74 years (Fig. 1A; Table 1).

**Table 1:** Numbers, participation-based rates, incidence rate ratios (IRRs), and *P* values for sports- and recreation-related deaths by natural causes, as categorized in the *Étude des blessures subies au cours de la pratique d’activités récréatives et sportives au Québec* (ÉBARS), and considering the populations surveyed in the ÉBARS in Québec, Canada, January 1, 2006, to December 31, 2019 (inclusive)

Activities†,∗	n	Participation year	Rate (95% CI)	Time-period comparison	IRR (95% CI)	*P*
Cycling	53	47,626,000	0.11 (0.08–0.15)	2 vs 1	0.63 (0.32–1.24)	0.1834
				3 vs 2	1.16 (0.59–2.29)	0.6584
				3 vs 1	0.74 (0.39–1.39)	0.3443
				2006–2019	1.00 (0.93–1.07)	0.9537
Ice hockey	24	12,714,000	0.19‡ (0.12–0.28)	2 vs 1	1.00 (0.39–2.53)	1.0000
				3 vs 2	0.54 (0.20–1.50)	0.2386
				3 vs 1	0.54 (0.19–1.57)	0.2598
				2006–2019	0.93 (0.84–1.03)	0.1648
Jogging/running	20	25,342,000	0.08‡ (0.05–0.12)	—	—	—
Hiking or taking a walk	18	63,383,000	0.03‡ (0.02–0.04)	—	—	—
Swimming	14	48,408,000	0.03‡ (0.02–0.05)	—	—	—
Physical conditioning§	12	37,063,000	0.03‡ (0.02–0.06)	—	—	—
All-terrain vehicle	8	18,802,000	F	—	—	—
Snowshoeing	8	19,801,000	F	—	—	—
Snowmobile	7	10,410,000	F	—	—	—
Golfing	7	12,619,000	F	—	—	—
Alpine skiing	6	14,175,000	F	—	—	—
Basketball‖	5	7,643,000	F	—	—	—
Cross-country skiing	4	10,505,000	F	—	—	—
Inline skating	3	12,409,000	F	—	—	—
Soccer	3	13,922,000	F	—	—	—
Nonmotorized navigation¶	3	17,290,000	F	—	—	—
Combat sports	2	5,095,000	F	—	—	—
Racket sports	2	16,634,000	F	—	—	—
Ice skating	1	27,077,000	F	—	—	—
Football‖	0	3,900,000	F	—	—	—
Skateboarding∗∗	0	3,959,000	F	—	—	—
Snowboarding	0	6,337,000	F	—	—	—
Baseball–softball	0	6,424,000	F	—	—	—
Volleyball‖	0	7,307,000	F	—	—	—
All activities††	200	86,295,137	0.23 (0.20–0.27)	2 vs 1	0.85 (0.60–1.20)	0.3572
				3 vs 2	0.95 (0.68–1.32)	0.7493
				3 vs 1	0.81 (0.57–1.13)	0.2131
				2006–2019	0.99 (0.95–1.02)	0.3923

Rates (numerator: number of deaths; denominator: ÉBARS participation data, considering only the activities listed in the table) are expressed as a number per 100,000 participant-years. The first 3 IRRs reflect the average change in incident rates between periods, and the P-values test the null hypothesis of no difference among IRRs among periods. The last IRR reflects the average annual change in incidence rates between consecutive years, and the P-value tests the null hypothesis of no linear trend in the incidence rate during the study period. F indicates a coefficient of variation > 33.3%. These rates are too unreliable to be reported.

CI, confidence interval.

∗ IRR of Activities with fewer than five deaths within a given time period (22 out of 24 activities) were not reported in this table.

† 6-74 years old.

‡ Coefficient of variation between 16.6% and 33.3%. These rates are to be interpreted with caution.

§ Includes activities such as step training, aerobics, strength training (including bodyweight exercises), circuit training, or combinations thereof. Also includes activities explicitly described as physical conditioning by the coroner without further details.

‖ Aged 6–64 years.

¶ Refers to activities where the participant propels the craft using physical effort rather than a motor.

∗∗ Aged 6–44 years.

†† Activities listed in the table only.

### All deaths: numbers and population-based rates

During the 14-year period, 297 natural deaths occurred that were associated with sport and recreation activities in Québec, averaging 21.2 deaths per year. The population-based death rate was 0.26 per 100,000 person-years (Table 2). Cycling (20.5%; n = 61; 0.05 per 100,000 person-years) and team sports (15.2%; n = 45; 0.04 per 100,000 person-years) accounted for the highest percentages of fatalities, with ice hockey (8.8%; n = 26; 0.02 per 100,000 person-years) accounting for the major proportion of the team sports incidents (Supplemental Table S1). Aside from cycling and ice hockey, the 4 activities with the next-highest frequencies of death were hunting (n = 24), jogging/running (n = 22), hiking or walking (n = 21), and swimming (n = 20). No significant differences occurred in general trends (2006-2019) in IRRs, for all activities (Table 2). Male participants (n = 275) represented 92.6% of all natural deaths. Death rates began to rise from age 35 across all age groups, with the highest rates observed in males aged 45 and over (Fig. 1B).

**Table 2:** Numbers, population-based rates, incidence rate ratios (IRRs), and P-values for all sports- and recreation-related deaths, by natural causes in Québec, Canada, January 1, 2006, to December 31, 2019 (inclusive)

Activities∗	n	%	Rate (95% CI)	Time-period comparison	IRR (95% CI)	*P*
Cycling	61	20.54	0.05 (0.04–0.07)	2 vs 1	0.69 (0.37–1.31)	0.2562
				3 vs 2	1.24 (0.67–2.29)	0.5003
				3 vs 1	0.85 (0.47–1.56)	0.6080
				2006–2019	1.02 (0.95–1.08)	0.6376
Team sports	45	15.15	0.04 (0.03–0.05)	2 vs 1	1.34 (0.66–2.73)	0.4133
				3 vs 2	0.55 (0.27–1.12)	0.1015
				3 vs 1	0.74 (0.33–1.65)	0.4676
				2006–2019	0.96 (0.89–1.03)	0.2817
Hiking and taking a walk or snowshoeing	31	10.44	0.03† (0.02–0.04)	2 vs 1	0.58 (0.20–1.66)	0.3074
				3 vs 2	2.74 (1.08–6.95)	0.0337∗∗
				3 vs 1	1.58 (0.68–3.66)	0.2863
				2006–2019	1.05 (0.96–1.15)	0.2557
Hunting	24	8.08	0.02† (0.01–0.03)	—	—	—
Jogging/running	22	7.41	0.02† (0.01–0.03)	—	—	—
Swimming and underwater activities	22	7.41	0.02† (0.01–0.03)	2 vs 1	0.38 (0.13–1.12)	0.0807
				3 vs 2	1.35 (0.43–4.27)	0.6043
				3 vs 1	0.52 (0.20–1.37)	0.1850
				2006–2019	0.95 (0.86–1.06)	0.3489
Land motor sports	21	7.07	0.02† (0.01–0.03)	2 vs 1	0.55 (0.17–1.73)	0.3055
				3 vs 2	1.74 (0.58–5.20)	0.3199
				3 vs 1	0.96 (0.36–2.57)	0.9284
				2006–2019	1.00 (0.90–1.11)	0.9939
Physical conditioning‡	16	5.39	0.01† (0.01–0.02)	—	—	—
Navigation§	13	4.38	0.01† (0.01–0.02)	—	—	—
Alpine skiing	9	3.03	F	—	—	—
Golfing	7	2.36	F	—	—	—
Cross-country skiing	6	2.02	F	—	—	—
Fishing (not in a boat)						
(not in a boat)	4	1.35	F	—	—	—
Skating (ice and inline)	4	1.35	F	—	—	—
Dancing	3	1.01	F	—	—	—
Combat sports	3	1.01	F	—	—	—
Racket sports	2	0.67	F	—	—	—
Undefined (physical education)‖	2	0.67	F	—	—	—
Equestrian activities	1	0.34	F	—	—	—
Paintball	1	0.34	F	—	—	—
All activities¶	297	100.00	0.26 (0.23–0.30)	2 vs 1	0.93 (0.70–1.23)	0.5913
				3 vs 2	0.94 (0.72–1.23)	0.6554
				3 vs 1	0.87 (0.65–1.16)	0.3376
				2006–2019	0.99 (0.96–1.02)	0.5181

Rates are expressed as a number per 100,000 person-years. The first 3 IRRs reflect the average change in incident rates between periods, and the P-values test the null hypothesis of no difference among IRRs among periods. The last IRR reflects the average annual change in incidence rates between consecutive years, and the P-value tests the null hypothesis of no linear trend in the incidence rate during the study period. F indicates a coefficient of variation > 33.3%. These rates are too unreliable to be reported.

CI, confidence interval.

∗ IRR of activities with < 5 deaths within a given time period (15 of 20 activities) were not reported in this table.

† Coefficient of variation between 16.6% and 33.3%. These rates are to be interpreted with caution.

‡ Includes activities such as step training, aerobics, strength training (including bodyweight exercises), circuit training, or combinations thereof; also includes activities explicitly described as physical conditioning by the coroner without further details.

§ Refers to both motorized and nonmotorized navigation activities, where nonmotorized activities involve propelling the craft using physical effort rather than a motor.

‖ The total number of physical education–related natural deaths is 3 (n = 3) because 1 cross country skiing–related death occurred in the context of a physical education class.

¶ For a detailed list of categories, underlying activities, and the specific numbers of unique sport and recreation activities linked to natural deaths, refer to Supplemental Table S1.

∗∗ *P* < 0.05.

### Etiology and characteristics of the fatalities

Sudden death accounted for 98.7% of natural deaths (n = 293) in sport and recreation, and 95.3% of all fatalities (n = 283) were of cardiac origin. The specific SCD (n = 281) rate was 0.25 per 100,000 person-years (95% confidence interval: 0.22-0.28). AEDs were not available at the death site in 65% of cases (n = 193), whereas bystander CPR was performed in 60.9% of all cases (n = 181). Most deaths (93.9%; n = 279) did not occur during an organized competition or event, and the deceased was alone at the time of the incident in 39.7% of cases (n = 118). A blood alcohol concentration > 80 mg/100 mL, or the presence of other potentially cardiotoxic substances, was observed in 1.7% (n = 5) and 8.4% (n = 25) of the cases, respectively (Table 3). Acute coronary syndrome was the main cause of death in these cases (n = 3 and n = 21, respectively)

**Table 3:** Number, population-based rates, and characteristics for sports- and recreation-related deaths by natural causes, in Québec, Canada, January 1, 2006, to December 31, 2019 (inclusive)

Characteristics and factors	n	%
**Cause of death**		
Cardiac	283	95.3
Noncardiac	13	4.4
Undetermined	1	0.3
**Sudden death**		
Yes	293	98.7
No	4	1.3
**Sudden cardiac death**		
Yes	281	94.6
No	16	5.4
**AEDs**		
Documented onsite AED utilized∗	35	11.8
Documented onsite AED not utilized	1	0.3
First-responder AED utilized	1	0.3
Absence of AED onsite and no first-responder AED utilized†	193	65.0
Unavailable information	67	22.6
**Bystander cardiopulmonary resuscitation**		
Yes	181	60.9
No	75	25.3
Unavailable information	41	13.8
**Organized competition or event**		
Yes	18	6.1
No	279	93.9
**Alone at the time of the incident**		
Yes	118	39.73
No	161	54.21
Unavailable information	18	6.06
**Alcohol consumption**		
Blood alcohol concentration ≤ 80 mg/100 mL (0.08)	10	3.4
Blood alcohol concentration > 80 mg/100 mL (0.08)	5	1.7
No	222	74.7
Unavailable information	60	20.2
**Positive for other potentially cardiotoxic substances**	**25**	**8.4**
Cannabis	8	2.7
Cocaine	10	3.4
Other drugs with potential effects on QT prolongation‡	16	5.4
No or unavailable information	272	91.6
**Place of occurrence**		
Sports complex	66	22.2
Road	49	16.5
Trail (marked or unmarked)	39	13.1
Forest or field	36	12.1
Pool (residential or public)	16	5.4
Cycling path	15	5.1
Lake	15	5.1
Residency	14	4.7
Ski resort	9	3.0
Other	38	12.8
**Region**		
Bas-Saint-Laurent	10	3.4
Saguenay–Lac-Saint-Jean	11	3.7
Capitale-Nationale	25	8.4
Mauricie	13	4.4
Estrie	26	8.8
Montréal	38	12.8
Outaouais	16	5.4
Abitibi-Témiscamingue	17	5.7
Côte-Nord	20	6.7
Nord-du-Québec	6	2.0
Gaspésie–Îles-de-la-Madeleine	8	2.7
Chaudière-Appalaches	13	4.4
Laval	8	2.7
Lanaudière	8	2.7
Laurentides	37	12.5
Montérégie	24	8.1
Centre-du-Québec	17	5.7

Rates are expressed as a number per 100,000 person-years. These rates are too unreliable to be reported.

Coefficient of variation between 16.6% and 33.3%. These rates are to be interpreted with caution.

AED, automated external defibrillator.

∗ Ice hockey (n = 9), alpine skiing (n = 4), jogging/running (n = 4), swimming (n = 3), and off-ice hockey (n = 3) together represented 65.7% of these cases.

† Cycling (n = 42), hunting (n = 18), hiking or walking (n = 18), jogging/running (n = 15), swimming (n = 11), and physical conditioning (n = 11) together represented 59.6% of these cases.

‡ Mostly antidepressant drugs.

The leading causes of death were acute coronary syndrome in adults aged ≥ 35 years (71.9%; n = 184), and malignant cardiac arrhythmia in individuals aged ≤ 34 years (63.4%; n = 26). Malignant cardiac arrhythmia was responsible for 91.7% of deaths (n = 11) in individuals aged ≤ 17 years. Across all age groups, cerebral hemorrhage was identified as the most common cause of noncardiac death, accounting for 3.0% (n = 9) of the total burden (Table 4). Comparison of cardiac causes of death between individuals aged ≥ 35 years and those aged ≤ 34 years revealed that acute coronary syndrome was more prevalent among adults aged ≥ 35 years (75.1% vs 28.9%, *P* < 0.0001), whereas malignant cardiac arrhythmia was more common in individuals aged ≤ 34 years (68.4% vs 22.4%, *P* < 0.0001).

**Table 4:** Causes of natural deaths associated with sports- and recreation-related activities by age groups, in Québec, Canada, January 1, 2006, to December 31, 2019 (inclusive)

			Age, y					
Causes	≤ 17		18–34		≥ 35		All adults (≥ 18)	
	n	%	n	%	n	%	n	%
**Cardiac**∗	**11**	**91.7**	**27**	**93.1**	**245**	**95.7**	**272**	**95.4**
Acute coronary syndrome	0	0	11	37.9	184	71.9	195	68.4
Malignant cardiac arrhythmia (without documented underlying genetic causes)	9	75	11	37.9	55	21.5	66	23.2
Malignant cardiac arrhythmia in a setting of hypertrophic cardiomyopathy	1	8.3	2	6.9	0	0.0	2	0.7
Malignant cardiac arrhythmia in a setting of right ventricular cardiomyopathy	1	8.3	2	6.9	0	0.0	2	0.7
Aortic aneurysm rupture	0	0	0	0	3	1.2	3	1.1
Aortic dissection	0	0	0	0	3	1.2	3	1.1
Aortic hypoplasia	0	0	1	3.4	0	0.0	1	0.4
**Noncardiac**	**1**	**8.3**	**2**	**6.9**	**10**	**3.9**	**12**	**4.2**
Cerebral hemorrhage	1	8.3	2	6.9	6	2.3	8	2.8
Pulmonary embolism	0	0	0	0	2	0.8	2	0.7
Secondary cerebral anoxia due to a convulsive seizure	0	0	0	0	1	0.4	1	0.4
Asthma exacerbation	0	0	0	0	1	0.4	1	0.4
**Undetermined**	**0**	**0**	**0**	**0**	**1**	**0.4**	**1**	**0.4**
**All**	**12**	**100**	**29**	**100**	**256**	**100**	**285**	**100**

The bold values indicate that the non-bold values/variables beneath them are included within.

∗ Significant differences between individuals aged ≥ 35 years and those aged ≤ 34 years for 3 clustered categories of cardiac causes (acute coronary syndrome, malignant cardiac arrhythmia, and aortic causes) were analyzed using χ^2^ tests. Significant differences were observed for acute coronary syndrome (*P* < 0.0001) and malignant cardiac arrhythmia (*P* < 0.0001), but not for aortic causes (*P* = 1.0000), for which a Fisher’s exact test was used, due to the low frequency of deaths. To control for the overall type I error rate, a global test was performed beforehand (*P* < 0.0001).

Among adults who died from cardiac causes, the mean age of the deceased was 53.9 ± 14.0 years, and SCD accounted for 99.3% (n = 270) of the fatalities (Table 5). At least 1 CVRF was recorded in 37.5% (n = 102) of the cases, as follows: obesity (n = 49); hypertension (n = 47); dyslipidemia (n = 34); diabetes mellitus (n = 15); smoking (n = 14); and positive family history (n = 13). Cardiac decedents had a mean BMI of 27.9 ± 4.8 kg/m^2^ (n = 133) and a mean cardiac weight of 475.2 ± 104.4 grams. The findings documented most frequently in autopsies of victims who died from cardiac-related causes include evidence of significant coronary atherosclerotic heart disease (n = 188) and aortic anomalies (n = 137), representing 69.1% and 50.4% of all adult cardiac deaths, respectively. The leading causes of natural death in adults with a cardiac weight > 500 grams were, respectively, acute coronary syndrome (58.5%; n = 38) and malignant cardiac arrhythmia (38.5%; n = 25; Supplemental Table S2).

**Table 5:** Characteristics of adult cardiac and noncardiac deaths associated with sport and recreation activities, in Québec, Canada, January 1, 2006, to December 31, 2019 (inclusive)

Characteristic	All deaths∗	Cardiac deaths	Noncardiac deaths
**Number of subjects**	284	272	12
Age, y	53.9 ± 14.2	53.9 ± 14.0	53.9 ± 17.7
Male	267 (94.0)	256 (94.1)	11 (91.7)
Age ≥ 35 y	255 (89.8)	245 (90.1)	10 (83.3)
Sudden death	280 (98.6)	270 (99.3)	10 (83.3)
Documented cardiovascular risk factors			
≥ 1	106 (37.3)	102 (37.5)	4 (33.3)
≥ 2	51 (18)	51 (18.8)	0 (0)
**Autopsy practiced**	227 (79.9)†	217 (79.8)†	10 (83.3)†
BMI, kg/m^2^	27.8 ± 4.8 (n = 139)¶	27.9 ± 4.8 (n = 133)	25.3 ± 4.8 (n = 6)
Cardiac weight documented	192 (84.6)‡	186 (85.7)‡	6 (60)‡
Cardiac weight, g	472.6 ± 105.5	475.2 ± 104.4	393.5 ± 118.3
Cardiac weight > 500 g	65 (33.9)§	64 (34.4)§	1 (16.7)§
LV wall thickness documented	130 (57.3)‡	126 (58.1)‡	4 (40)‡
LV wall thickness, mm	16 ± 0.4	16 ± 0.4	18 ± 0.8
LV wall thickness > 15 mm	71 (54.6)§	70 (55.6)§	1 (25)§
Septal thickness documented	71 (31.7)‡	71 (32.7)‡	1 (10)
Septal thickness, mm	16 ± 0.3	16 ± 0.3	15
**Presence or absence documented**			
Fibrosis	60 (26.4)‡	58 (26.7)‡	2 (20)‡
Evidence present	54 (90)§	53 (91.4)§	1 (50)
Valvular involvement	63 (27.8)‡	61 (28.1)‡	2 (20)‡
Evidence present	6 (9.5)§	6 (9.8)§	0 (0)§
Malposition of the coronary arteries	6 (2.6)‡	6 (2.8)‡	0 (0)‡
Evidence present	2 (33)§	2 (33)§	0 (0)§
Coronary calcifications	18 (7.9)‡	17 (8.3)‡	1 (10)‡
Evidence present	16 (88.9)§	15 (88.2)§	1 (100)§
Coronary atherosclerotic heart disease	215 (94.7)‡	207 (95.4)‡	8 (80)‡
Evidence present	194 (90.2)§	188 (90.8)§	6 (75)§
Aorta anomaly	143 (63)‡	139 (64.1)‡	4 (40)‡
Evidence present	141 (98.6)§	137 (98.6)§	4 (100)§
Aortic calcium score (grade)‖	2.6 ± 1.2	2.6 ± 1.2	2.5 ± 1

Values are given as n, mean ± standard deviation, or n (%).

BMI, body mass index; LV, left ventricular.

∗ Excluding 1 natural death of undetermined cause.

† Although an autopsy was practiced in 79.9% of adult patients, multiple data were missing in the reports.

‡ Percentage was based on the number of autopsies practiced on adult patients for a given category: all deaths (n = 227); cardiac deaths (n = 217); and noncardiac deaths (n = 10).

§ Percentage based on the number of cases with data available for a given variable. For instance: 65 (cardiac weight > 500 g for all deaths) / 192 (cases with data available on cardiac weight for all deaths) ∗ 100 = 33.9%.

‖ When 2 different scores were documented for different segments of the aorta by the pathologist, the highest score was recorded.

¶ BMI data were available in 133 of the 227 adult autopsy reports. For the remaining 6 cases, BMI information was sourced from the coroner or police reports, totaling 139 for which BMI data were available.

Among the pediatric population who died from cardiac causes, the mean age of the deceased was 13.8 ± 3.5 years, and SCD represented 100% of the deaths (n = 11; Table 6). Cardiac decedents had a mean BMI of 27.5 ± 10 kg/m^2^ (n = 5).

**Table 6:** Characteristics of pediatric cardiac and noncardiac deaths associated with sport and recreation, in Québec, Canada, January 1, 2006, to December 31, 2019 (inclusive)

Characteristics	All deaths	Cardiac deaths
**Subjects**	12	11
Age, y	14.1 ± 3.4	13.8 ± 3.5
Male	8 (66.7)	7 (63.6)
Sudden death	12 (100)	11 (100)
**Autopsy practiced**	12 (100)∗	11 (100)∗
Body mass index, kg/m^2^	27.5 ± 10 (n = 5)	27.5 ± 10 (n = 5)
Cardiac weight documented	10 (83.3)†	9 (81.8)†
Cardiac weight, g	390.8 ± 134.9	387.6 ± 142.7
Cardiac weight > 500 g	2 (20)‡	2 (22.2)‡
LV wall thickness documented	6 (50.0)†	6 (54.5)†
LV wall thickness, mm	14 ± 0.3	14 ± 0.3
LV wall thickness > 15 mm	1 (16.7)‡	1 (16.7)‡
Septal thickness documented	5 (41.7)†	5 (45.5)†
Septal thickness, mm	16 ± 0.4 (n = 5)	16 ± 0.4 (n = 5)
**Presence or absence documented**		
Fibrosis	3 (25)†	3 (25)†
Evidence present	2 (66.7)‡	2 (66.7)‡
Valvular involvement	4 (40)†	4 (40)†
Evidence present	1 (25)‡	1 (25)‡
Malposition of the coronary arteries	2 (16.7)†	2 (18.2)†
Evidence present	2 (100)‡	2 (100)‡
Coronary calcifications	0 (0)†	0 (0)†
Evidence present	0 (0)‡	0 (0)‡
Availability of data on atherosclerotic heart disease	6 (50)†	6 (54.5)†
Evidence of coronary atherosclerotic heart disease	2 (33.3)‡	2 (33.3)‡
Aorta anomaly	2 (16.7)†	2 (18.2)†
Evidence present	0 (0)‡	0 (0)‡
Aortic calcium score (mean grade)§	n/a	n/a

Values are given as n, mean ± standard deviation, or n (%).

n/a, not applicable.

∗ Although an autopsy was practiced for all pediatric patients, multiple data were missing in the reports.

† Percentage based on the number of autopsies practised on pediatric patients for a given category: all deaths (n = 12); cardiac deaths (n = 11); and noncardiac deaths (n = 1).

‡ Percentage based on the number of cases with data available for a given variable. For instance, 2 (cardiac weight > 500 g for all deaths) / 10 (cases with data available on cardiac weight for all pediatric deaths) ∗ 100 = 20%.

§ When 2 different scores were documented for different segments of the aorta by the pathologist, the highest score was recorded.

Female participants accounted for 7.4% of all natural deaths (n = 22) in sport and recreation, with all cases classified as sudden deaths. Among these, swimming (n = 7) and hiking or walking (n = 4) accounted for half of the cases. The mean age of the deceased was 48.4 ± 22.9 years, and the mean BMI was 26.9 ± 4.5 kg/m^2^ (n = 12). The majority, 90.1% (n = 20), died of cardiac causes, including coronary syndrome (n = 13) and malignant arrhythmias (n = 7).

## Discussion

This study analyzed trends in the frequencies and rates of natural deaths associated with sport and recreation in Québec over a 14-year period, and investigated the causes and characteristics of these fatalities. As part of a global research project on sports- and recreation-related deaths, encompassing both unintentional injury deaths and deaths from natural causes, this study provides a unique and comprehensive perspective. Notably, natural deaths accounted for 13.3% of all sport and recreation-related deaths, highlighting the significance of our findings and their relative importance within the global context.^5^ Although previous studies often have focused solely on athletes,^11^^,^^29^ this investigation is one of the few to include both pediatric and adult individuals from the general population.^2^^,^^4^^,^^7^^,^^8^^,^^10^^,^^30^, ^31^, ^32^ In addition, this study stands out, owing to its comprehensive examination of all causes of natural deaths related to sport and recreation, even though SCD constitutes a significant majority of these occurrences.^4^ The population-based death rates for natural deaths (0.26 per 100,000 person-years) and SCD (0.25 per 100,000 person-years) that are related to sport and recreation in the province of Québec align with the rates observed in comparable population studies (SCD rates, per 100,000 person-years) conducted in Germany (0.12), Spain (0.38), France (0.46), and Australia (0.5-0.98).^2^^,^^7^^,^^8^^,^^30^ Male individuals represented a majority (92.6%) of all natural deaths, a percentage that also is consistent with these studies.^2^^,^^7^^,^^8^^,^^30^

In the current study, bystander CPR was performed in 60.9% of all cases, a rate that is encouraging, compared to that in France, where CPR was initiated in 30.7% of sports-related sudden deaths in the general population.^2^ The high rate we observed is particularly significant given that CPR greatly enhances survival rates—victims are nearly 4 times more likely to survive a cardiac arrest if a bystander performs CPR.^33^^,^^34^ Data from various Canadian provinces (Alberta, British Columbia, Nova Scotia, and Ontario) showed bystander CPR rates ranging from 14.7% to 46.0%,^35^ underscoring the strength of the rates observed in this study. An international systematic review analyzing bystander interventions, in incidences of exercise-related sudden cardiac arrest, reported that bystander CPR was administered at a median rate of 71% (interquartile range: 59%-87%).^36^ However, these rates are expected to be higher, compared to those observed in the current investigation, as the latter included only those cases resulting in death.

Interesting data show that, following the implementation of legislation in China that focused on deploying AEDs and providing public CPR training in low- and middle-income settings, a significant increase was observed in bystander CPR rates, and an improved survival rate of patients with out-of-hospital cardiac arrest.^37^ Similarly, in Victoria, Australia, regions with lower CPR training rates were associated with both lower bystander CPR rates and diminished survival outcomes.^38^ These findings suggest that a legislative approach could be beneficial for further enhancing survival rates in Québec and improving outcomes in remote and underserved areas. However, the incidence of administration of a shock by an AED was higher in the French, sports-related study, at 61%,^2^ significantly surpassing the present findings showing that AEDs were utilized in only 12% of cases, including onsite and first-responder AEDs. The lack of AED utilization in most cases of this study is concerning, given the findings from studies in the Netherlands. On one hand, AED use has been associated with higher survival rates in patients with a shockable initial rhythm.^39^ On the other hand, neurologically intact survival rates were 49.6% for patients treated with onsite AEDs, 17.2% for those treated with dispatched AEDs, and 14.3% for those with no AED use.^40^ Similarly, a systematic review and meta-analysis of studies from Australia, Canada, Japan, the Netherlands, and the US found that AED use was linked to increased rates of survival to hospital discharge, and improved neurologic outcomes.^41^ Besides, throughout the study period, nearly 60% of coroners’ recommendations related to natural deaths in sport and recreation in the province of Québec emphasized AED implementation, delivery, and training.^13^ Despite these recommendations and efforts to increase AED availability, no significant reduction in natural death rates related to sport and recreation was observed over the study period. This finding highlights the urgent need for more robust and integrated strategies, such as legislation, to address the mortality risks in these settings.

The primary cause of natural death in adults aged ≥ 35 years (71.9%) was acute coronary syndrome, consistent with findings from other studies.^2^^,^^7^^,^^30^ This finding is supported further by the documented presence of significant coronary atherosclerotic heart disease in 69.1% of all adult cardiac deaths. Notably, the incidence of coronary atherosclerotic heart disease increases with age and has been identified as the most prevalent finding among victims aged ≥ 35 years in previous studies.^10^^,^^42^, ^43^, ^44^ Moreover, the recording of CVRFs in 37.5% of adult natural deaths is noteworthy, especially given the lack of systematic documentation in coroner reports and autopsy records. This finding highlights the critical need to standardize data-recording practices in autopsy reports in Québec and to implement systematic medical screening for individuals at risk of CVRF-related conditions. A systematic cardiac screening for male individuals aged ≥ 35 years may be contemplated in the province, akin to the Québec Breast Cancer Screening Program that targets women aged 50-74 years. However, the shortage of family doctors in the province of Québec who are available to assess individuals’ risk and symptoms before the individuals engage in physical activities is particularly concerning, especially for elderly and sedentary individuals, who may benefit from early intervention and preventive measures. This issue extends beyond Québec, highlighting the need for nationwide reflection, as millions of Canadians lack access to a family doctor. Limited resources and inadequate infrastructure for screening are significant barriers that must be addressed through policy changes and increased funding. In Canada, online screening portals with validated questionnaires, such as the Community & Athletic Cardiovascular Health (CATCH) Network screening portal, are available for healthcare professionals.^45^ This portal currently is accessible to sports organizations and institutions interested in incorporating cardiovascular screening into their programs and patient care.

Malignant cardiac arrhythmia emerged as the leading cause of natural death in individuals aged ≤ 34 years (in 63.4%), with an even higher proportion observed among those aged ≤ 17 years (91.7%). Additionally, cardiomyopathies were observed exclusively in individuals aged ≤ 34 years, and all pediatric cardiac deaths were attributed to SCD. These findings are consistent with other research results indicating that arrhythmias and inherited cardiomyopathies occur predominantly in younger cohorts.^7^^,^^46^ Therefore, consideration of familial predisposition, particularly toward SCD, is essential in developing a proactive approach to preventing natural deaths related to sport and recreation.^15^, ^16^, ^17^, ^18^ Regrettably, the genetic diagnosis of arrhythmogenic right ventricular cardiomyopathy and/or dysplasia, which could be valuable for family screening and timely intervention,^21^^,^^47^^,^^48^ was not documented in the autopsy reports consulted for this study. This absence limits opportunities for affected relatives to benefit from appropriate management and lifestyle modifications.^21^ Although hereditary factors are considered and discussed by coroners who engage directly with the family of the deceased, establishing protocols that ensure routine genetic testing during autopsies of SCD victims is imperative. These genetic tests should be conducted systematically for individuals with hereditary cardiomyopathy, and for those aged ≤ 34 years with no significant atherosclerosis or other identifiable causes of death in the context of SCD. Implementing such procedures would significantly enhance familial screening and intervention strategies.^19^^,^^20^

The democratization of sport and recreation, and the popularity of unstructured activities, should be emphasized. Remarkably, 93.9% of the deaths occurred outside of organized competitions or events. Only 22% of deaths took place in sports complexes, which is a relatively low percentage, compared to those in French studies in which more than half of sports-related sudden deaths occurred in facilities such as gyms or stadiums.^2^^,^^32^ Moreover, 5 of the 6 activities with the highest frequencies of deaths (cycling, hunting, jogging and/or running, hiking or walking, and swimming; n = 148, representing half of all natural deaths in sport and recreation contexts) commonly are practiced in locations where AEDs are not readily available. Therefore, although concerning, it is not surprising that AEDs were unavailable at the death site in 65% of all cases in this study. Although this study focused on Québec, this situation likely is applicable to other Canadian provinces, where people are dispersed over large areas and engage in informally organized activities in remote settings. This situation mirrors that in Spain, where over half of sports-related sudden deaths occurred outside sports venues, revealing a deficiency in AED availability.^8^ Enhancing medical screening for populations engaged in sport and recreation (and other physically demanding tasks) in remote areas, such as hunting, may be a focal point for intervention. In addition, considering the substantial challenges, regarding accessibility, encountered by first responders in remote or rural areas,^49^ innovative solutions, such as portable AEDs or an optimized drone network to expedite AED delivery, merit serious consideration.^50^^,^^51^ These proactive and imaginative initiatives aimed at ensuring AED availability, complemented by comprehensive CPR training for the public, are vital for enhancing emergency-response capabilities in such contexts, aiming to ultimately prevent avoidable deaths effectively. Notably, half of the deceased in these aforementioned activities (n = 76 of 148; data not shown) were alone at the time of the incident. Hence, accompaniment emerges as a fundamental complementary preventive measure, alongside AED- and CPR-based strategies. Additionally, community-based interventions suitable for resource-limited settings should be considered to optimize survival outcomes in sports- and recreation-related incidents that occur in remote areas. These interventions include community-responder networks, basic life-support training, regional resuscitation campaigns, crowdsourcing technologies, and dispatcher-assisted CPR programs.^52^

Although addressing the challenges in remote areas is important, the implementation of AED installation in heavily frequented public venues is also crucial. In 2013, the Canadian government initiated a program to equip all hockey arenas nationwide with AEDs. Over the analyzed period, devices also were deployed in fitness centres, gyms, and public pools. However, the uniformity of AED distribution was hampered by the absence of AED-related legislation in the province of Québec as of 2024. This legislative gap had a significant impact, as the province was ranked second to last in AED availability, having only 27 AEDs per 100,000 inhabitants, based on a 2019-2020 Canadian survey.^53^ In contrast, Manitoba emerged as a leader among the surveyed provinces, boasting 324 AEDs per 100,000 inhabitants, which is more than 10 times the number in Québec. This striking gap can be attributed directly to The Defibrillator Public Access Act, implemented in Manitoba in 2013, which mandates AED placement in heavily frequented public venues.^54^ Following Manitoba's leadership, the Ontario government enacted similar legislation in June 2020.^55^ The province of Québec adopted a comprehensive deployment strategy in January 2023, aiming to install 1000 AEDs across the province. As part of this strategy, 100 AEDs were installed in financial-institution branches, specifically targeting rural areas for enhanced coverage. By July 2024, more than 7000 AEDs located in public areas were registered in the AED—Québec database, highlighting progress toward broader accessibility driven by strategic governmental intervention. However, the impacts of these interventions in the province of Québec most likely are not as far-reaching and effective as those achieved through legislative mandates, as seen in provinces such as Manitoba and Ontario.

Furthermore, bolstering of efforts in complementary CPR training is essential. Starting in 2017, CPR training became an integral part of the academic curriculum, and a mandatory requirement for secondary school students (the Secondary III level, with students aged approximately 14 years), in Québec.^56^ This policy reflects a broader international trend observed since 2003, in which various countries and states have enacted legislation mandating CPR and AED education.^57^^,^^58^ Therefore, adopting a comprehensive approach (supported by legislation) aimed at AED accessibility, implementation support, training, and monitoring likely would contribute significantly to saving lives by enhancing emergency-response capabilities across the province.^58^^,^^59^

### Strengths, limitations, and perspectives

Considering that this study builds upon previous research, its inherent strengths and limitations were detailed extensively in a prior publication.^5^ Notable strengths encompass the comprehensive nature of the CD-BCQ database, the rigorous data-collection process, the utilization of 2 distinct denominators,^60^ and the meticulous data-entry procedures. These procedures were aligned specifically with the needs and characteristics of the at-risk population within the ÉBARS, ensuring precise and congruent numerator values for participation-based rates. Limitations included the fact that cases may not have been identified by the BCQ algorithms, particularly those related to emerging activities. Moreover, the coding of cases by the BCQ was subjective in nature, and the multiple-investigator data processing (which are both crucial components of the process and ultimately shape the definition of sport and recreation-related deaths in this study), while limitations, also contribute to the richness and detail of the information available, offering a rare and in-depth perspective on natural deaths that are related to sport and recreation in real life. Besides, for the period covered by the study, approximately 7.5% of all deaths in Québec were subject to a coroner’s investigation. Additionally, while autopsies were conducted in only 50% of all natural-death cases in Québec, the autopsy rate was higher for natural deaths that were specifically related to sport and recreation, with reports available for 80.5% of these cases. Consequently, some cases may not have been reported or investigated, particularly if the deceased was elderly or had a significant medical-history burden.

This study did not account for variations in the frequency of exposure within the participation data, and the presence of gaps in the years covered by participation data may have affected the accuracy of participation-based rates. Furthermore, some specific activities, age groups, and populations were not included in the ÉBARS surveys.

Other limitations included the inability to gather data on the participants' general training parameters (frequency, volume, intensity), for activities practiced before the incidents and for the incident itself. This information could have helped assess the relative risk level.^8^ The definition used for SCD in this study, which includes a 24-hour window from cessation of activity, differs from the standard 1-hour criterion used in most literature on SCD in sports. Although broad comparisons are possible, due to the similarities between cases and clinical situations, the difference in definitions limits the ability to draw accurate definitive interpretations.

Future studies with flexible definition parameters are needed to provide further insight and enable more-meaningful comparisons with our findings regarding natural death in unstructured sport and recreation contexts. Besides, the absence of standardized procedures and reporting practices in autopsies in the province of Québec has resulted in significant variability in the available autopsy-related information. For instance, basic information, such as BMI, was reported in only 57.7% of the 239 autopsy reports (n = 138). Only 2.9% of autopsy reports (n = 7) documented the presence or absence of fatty infiltration in the myocardium, and information related to genetic testing was available in only 2.1% of the reports (n = 5, data not shown). The documentation of the medical history of those deceased (sourced from autopsy and coroner reports) and CVRFs also was highly variable, potentially leading to the underreporting of certain CVRFs, as observed eleswhere.^7^ Moreover, the presumed cause of death likely induces a selection bias in the variables documented by pathologists in autopsy reports, highlighting the necessity for standardized practices.

Another challenge that emerged was due to the overlap in diagnoses of hypertrophic cardiomyopathy and isolated left-ventricular hypertrophy. In some instances, the available data did not allow for a definitive classification of these cases.^10^ Furthermore, instances in which data on CPR and AEDs were absent from police and coroner reports (documented as "unavailable information" in Table 3) likely suggest that CPR was not performed, and an AED was unavailable, potentially resulting in an underestimation of these deficiencies. The results of this study, and their practical applications—including identification of at-risk populations, the addressing of challenges in remote areas, and emphasizing the importance of medical screening, AEDs, CPR, and legislation—likely are transferable to other physically demanding tasks common in Canada, such as snow-shoveling and gardening. In addition to these secondary prevention measures, primary prevention strategies aimed at maximizing physical activity and sports participation in the general population are crucial for reducing cardiovascular risk^1^ and mitigating the likelihood of natural death, including SCD, during sport and recreation activities.

### Conclusion

In summary, this study underscores that most of the natural deaths associated with sport and recreation in Québec were cardiovascular-related, predominantly affecting males aged 45 and older. Despite the heightened availability of AEDs, mortality rates remained unchanged throughout the study period, underscoring the imperative for a comprehensive and contextually adapted approach. The frequent absence of AEDs at death sites highlights a significant gap in emergency-response capabilities. Proactive screening for CVRFs, and widespread deployment of AEDs at recreational sites, including remote areas, are essential steps to address these challenges. Legislative measures, as observed in other provinces, could catalyze AED installation implementation. By integrating risk assessment, ensuring AED availability and training, and improving emergency-response protocols, comprehensive strategies can be implemented to enhance safety and effectively reduce the incidence of sports- and recreation-related natural deaths in the province of Québec.
