# Supplementary material for: Natural Causes of Sports- and Recreation-Related Deaths in the General Population: A 14-Year Review in Québec, Canada
Source: CJC Open. 2024 Sep 10;7(1):53–66. doi: 10.1016/j.cjco.2024.09.002 (PMC11763571; doi:10.1016/j.cjco.2024.09.002)
Supplement: Supplemental Tables [file mmc1.pdf]

SUPPLEMENTAL TABLE S1

Number and Population-adjusted Rates for All Sport and Recreation-Related Deaths by Natural Causes, in Québec, Canada, January 1, 2006, to December 31, 2019 (Inclusive)*

Categories	n§	Rate (95%CI)	Activities	n§	Rate (95%CI)
Land motor sports	21	0.02++ (0.01-0.03)	All-terrain vehicle	9	F
			Snowmobile	10	0.01++ (0.00-0.02)
			Motocross	1	F
			Closed circuit racing	1	F
Swimming and underwater activities	22	0.02++ (0.01-0.03)	Swimming	20	0.02++ (0.01-0.03)
			Diving (scuba or snorkelling)	2	F
Cycling	61	0.05 (0.04-0.07)	No particularity or unspecified	57	0.05 (0.04-0.07)
			Mountain or fat biking	3	F
			BMX	1	F
Navigation	13	0.01++ (0.01-0.02)	Motorized	7	F
			Non-motorized	4	F
			Unspecified (motorized or not)	2	F
Hiking and taking a walk or snowshoeing	31	0.03++ (0.02-0.04)	Hiking and taking a walk	21	0.02++ (0.01-0.03)
			Snowshoeing	10	0.01++ (0.00-0.02)
Fishing	4	F	From the shore or a wharf	3	F
			In the water	1	F
Hunting and gun shooting	24	0.02++ (0.01-0.03)	Hunting	24	0.02++ (0.01-0.03)
			Gun shooting	0	F
Alpine skiing and snowboarding	9	F	Alpine skiing	9	F
			Snowboarding	0	F
Aerial activities	0	F	Non-motorized	0	F
			Motorized	0	F
Skateboarding	0	F	Skateboard or unspecified	0	F
			Longboard	0	F
Team sports	45	0.04 (0.03-0.05)	Ice hockey	26	0.02++ (0.02-0.03)

			Off-ice hockey	9	F
			Basketball	5	F
			Soccer	4	F
			Volleyball	1	F
Snow gliding	0	F	-	-	-
Jogging/running	22	0.02⁺⁺ (0.01-0.03)	-	-	-
Climbing	0	F	-	-	-
Skating	4	F	Inline skating	3	F
			Ice skating	1	F
Equestrian activities	1	F	Horse riding	0	F
			Cart or carriage	1	F
Physical conditioning	16	0.01⁺⁺ (0.01-0.02)	-	-	-
Combat sports	3	F	Boxing	2	F
			Kick-boxing or Muay Thai	1	F
Racket sports	2	F	Tennis	1	F
			Badminton	1	F
Dancing	3	F	-	-	-
Golfing	7	F	-	-	-
Cross country skiing	6	F	-	-	-
Other activities	3	F	Other outdoor games or activities: Paintball	1	F
			Undefined (Physical Education)[&]	2	F
All activities	297	0.26 (0.23-0.30)	-	-	-

*Rates are expressed as a number per 100 000 person-years.

[++] Coefficient of variation between 16.6% and 33.3%. These rates are to be interpreted with caution.

F Coefficient of variation > 33.3%. These rates are too unreliable to be reported.

[§]Zero death by natural causes reported (*n*=0) for activities in which unintentional injury deaths associated with sport and recreation (n≥1) have been recorded during the period.

[&]The total number of physical education-related natural deaths is three (n=3) since one cross country-skiing-related death occurred in the context of a physical education class.

SUPPLEMENTAL TABLE S2

Causes of Natural Deaths Associated with Sport and Recreation in Adults with Cardiac Weight > 500 g

	n	%	Mean cardiac weight (g)
Acute coronary syndrome	38	58.5	587 ± 100.2
Malignant cardiac arrhythmia (without documented underlying genetic causes)	23	35.4	575 ± 75.2
Malignant cardiac arrhythmia in a setting of hypertrophic cardiomyopathy	1	1.5	540
Malignant cardiac arrhythmia in a setting of right ventricular cardiomyopathy	1	1.5	518
Aortic aneurysm rupture	1	1.5	570
Pulmonary embolism	1	1.5	520
**All causes**	**65**	**100**	**580 ± 89.1**

SUPPLEMENTAL TABLE S3

Number and Participation-Adjusted Rates for Sport and Recreation-Related Deaths by Natural Causes, Considering the ÉBARS Surveyed Populations (6-74 years old), in Québec, Canada, January 1, 2006, to December 31, 2019 (Inclusive)*

Age	Sex	n	participation year (ÉBARS)	Rate (95%CI)	CV (%)	Rate (95%CI)
6-11	Male	1	3684000	0.03 (0.00-	100	F
6-11	Female	0	3534000	-	.	F
6-11	All	1	7213000	0.01 (0.00-	100	F
12-17	Male	5	3798000	0.13 (0.04-	44.7	F
12-17	Female	2	3667000	0.05 (0.01-	70.7	F
12-17	All	7	7455000	0.09 (0.04-	37.8	F
18-24	Male	7	4561000	0.15 (0.06-	37.8	F
18-24	Female	1	4427000	0.02 (0.00-	100	F
18-24	All	8	8993000	0.09 (0.04-	35.4	F
25-34	Male	11	6626687	0.17 (0.08-	30.2	0.17^{++} (0.08-0.30)
25-34	Female	0	6683981	-	.	F
25-34	All	11	13373000	0.08 (0.04-	30.2	0.08^{++} (0.04-0.15)
35-44	Male	27	7171232	0.38 (0.25-	19.2	0.38^{++} (0.25-0.55)
35-44	Female	3	7221698	0.04 (0.01-	57.7	F
35-44	All	30	14392925	0.21 (0.14-	18.3	0.21^{++} (0.14-0.30)
45-54	Male	47	7473477	0.63 (0.46-	14.6	0.63 (0.46-0.84)
45-54	Female	3	7402607	0.04 (0.01-	57.7	F
45-54	All	50	14876084	0.34 (0.25-	14.1	0.34 (0.25-0.44)
55-64	Male	56	6272925	0.89 (0.67-	13.4	0.89 (0.67-1.16)
55-64	Female	3	6423685	0.05 (0.01-	57.7	F
55-64	All	59	12696615	0.46 (0.35-	13	0.46 (0.35-0.60)
65-74	Male	31	3680880	0.84 (0.57-	18	0.84^{++} (0.57-1.20)
65-74	Female	3	4013471	0.07 (0.02-	57.7	F
65-74	All	34	7694346	0.44 (0.31-	17.1	0.44^{++} (0.31-0.62)

*Rates are expressed as number per 100,000 person per year

$^{++}$Coefficient of variation between 16.6% and 33.3%. These rates are to be interpreted with caution.

F Coefficient of variation > 33.3%. These rates are too unreliable to be reported.

SUPPLEMENTAL TABLE S4

Number and Population-based Rates for Sport and Recreation-Related Deaths by Natural Causes, in Québec, Canada, January 1, 2006, to December 31, 2019 (Inclusive)*

Age	Sex	n	Population	Rate (95%CI)	CV (%)	Rate (95%CI)
< 5	Male	0	3653736	-	.	F
≤ 5	Female	1	3496200	0.03 (0.00-	100	F
≤ 5	All	1	7149936	0.01 (0.00-	100	F
6-11	Male	1	3565998	0.03 (0.00-	100	F
6-11	Female	0	3416320	-	.	F
6-11	All	1	6982318	0.01 (0.00-	100	F
12-17	Male	7	3783487	0.19 (0.07-	37.8	F
12-17	Female	3	3635802	0.08 (0.02-	57.7	F
12-17	All	10	7419289	0.13 (0.06-	31.6	0.13[++] (0.06-0.25)
18-24	Male	12	5038162	0.24 (0.12-	28.9	0.24[++] (0.12-0.42)
18-24	Female	1	4813952	0.02 (0.00-	100	F
18-24	All	13	9852114	0.13 (0.07-	27.7	0.13[++] (0.07-0.23)
25-34	Male	15	7626694	0.20 (0.11-	25.8	0.20[++] (0.11-0.32)
25-34	Female	1	7310675	0.01 (0.00-	100	F
25-34	All	16	14937369	0.11 (0.06-	25	0.11[++] (0.06-0.17)
35-44	Male	37	7773730	0.48 (0.34-	16.4	0.48 (0.34-0.66)
35-44	Female	3	7494497	0.04 (0.01-	57.7	F
35-44	All	40	15268227	0.26 (0.19-	15.8	0.26 (0.19-0.36)
45-54	Male	66	8557835	0.77 (0.60-	12.3	0.77 (0.60-0.98)
45-54	Female	4	8432886	0.05 (0.01-	50	F
45-54	All	70	16990721	0.41 (0.32-	12	0.41 (0.32-0.52)
55-64	Male	78	7757442	1.01 (0.79-	11.3	1.01 (0.79-1.25)
55-64	Female	3	7826425	0.04 (0.01-	57.7	F
55-64	All	81	15583867	0.52 (0.41-	11.1	0.52 (0.41-0.65)
≥ 65	Male	59	8290283	0.71 (0.54-	13	0.71 (0.54-0.92)
≥ 65	Female	6	10320848	0.06 (0.02-	40.8	F
≥ 65	All	65	18611131	0.35 (0.27-	12.4	0.35 (0.27-0.45)

*Rates are expressed as number per 100,000 person per year

[++]Coefficient of variation between 16.6% and 33.3%. These rates are to be interpreted with caution.

F Coefficient of variation > 33.3%. These rates are too unreliable to be reported.
